# Association of the ICH Score With Withdrawal of Life‐Sustaining Treatment Over a 10‐Year Period

**DOI:** 10.1002/acn3.70136

**Published:** 2025-07-02

**Authors:** Nina Massad, Lili Zhou, Brian Manolovitz, Negar Asdaghi, Hannah Gardener, Hao Ying, Carolina M. Gutierrez, Angus Jameson, David Rose, Mohan Kottapally, Amedeo Merenda, Kristine O'Phelan, Sebastian Koch, Jose G. Romano, Tatjana Rundek, Ayham Alkhachroum

**Affiliations:** ^1^ Department of Neurology University of Miami Miller School of Medicine Miami Florida USA; ^2^ Department of Emergency Medicine Morsani College of Medicine, University of South Florida Tampa Florida USA; ^3^ Department of Neurology Morsani College of Medicine, University of South Florida Tampa Florida USA

**Keywords:** intracerebral hemorrhage, prognosis, withdrawal of life sustaining treatment

## Abstract

**Objective:**

The intracerebral hemorrhage (ICH) score was developed to enhance provider communication and facilitate early severity assessment. We examined the association of the ICH score with mortality and withdrawal of life‐sustaining treatment (WLST) in a large, multicenter stroke registry, and evaluated temporal trends in these associations.

**Methods:**

We identified ICH patients from the Florida Stroke Registry from 2013 to 2022. Outcomes were WLST and in‐hospital mortality. ICH scores were grouped as 0–2, 3–4, and 5–6. Importance plots identified key predictors of WLST. Model performance was assessed using AUC‐ROC for logistic regression and random forest, adjusted for relevant confounders. Secondary analyses compared outcomes between 2015–2018 and 2019–2022 using stratified univariate logistic regression.

**Results:**

In total, 12,426 patients were included (mean age 69, 55% male, 56% white). The most predictive factors associated with WLST were ICH score, age, state region, presenting level of consciousness, insurance status, and race (RF AUC = 0.94, LR AUC = 0.82). Mortality was 6.6%, 41.5%, and 66% for ICH score 0–2, 3–4, and 5–6. WLST occurred more frequently in higher ICH score groups (OR 9.35 [95% CI: 8.5–10.3] for scores 3–4; OR 18.64 [95% CI: 15.28–22.74] for scores 5–6). Early WLST (< 48 h) was more common in higher score groups (OR 2.97 [95% CI: 2.48–3.55] for 3–4; OR 9.51 [95% CI: 7.33–12.35] for 5–6).

**Interpretation:**

Higher ICH scores were strongly associated with mortality and WLST, including early withdrawal decisions. These associations remained largely consistent over time. These observational findings underscore the need for continued attention to how prognostic scores may influence WLST decisions.

## 1 Introduction

Intracerebral hemorrhage (ICH) has been consistently demonstrated to have higher morbidity and mortality risk than subarachnoid hemorrhage (SAH) or ischemic stroke [1, 2, 3]. The decision to withdraw life‐sustaining treatment (WLST) after ICH is common [2, 4, 5]. Mortality in ICH and other acute brain injuries has also been shown to be heavily mediated by WLST decisions [4, 6, 7].

Like other conditions within neurocritical care, ICH has seen the development of multiple clinical grading scores to aid outcome prediction [8, 9, 10]. However, concerns have been raised about overreliance on these tools in early decision making [11, 12]. One such tool is the ICH score, a clinical grading scale developed both as an outcome risk stratification scale and as a tool for providers to standardize ICH severity and aid inter‐provider communication [13]. The score incorporates factors including age, hematoma volume, location, presence of intraventricular hemorrhage (IVH) and Glasgow coma scale (GCS), totaling a score from zero to six. When initially described, an association was demonstrated between higher ICH score and outcome, defined as 30‐day mortality; however, its use as a tool predictive of outcome was cautioned [13].

Our study aimed to investigate the association between initial ICH score and WLST decisions, including early WLST (defined as WLST occurring within 48 h of hospital presentation), as well as in‐hospital mortality. We hypothesized that higher ICH scores would be associated with increased likelihood of WLST and early WLST. Additionally, we hypothesized that, over the study period, there would be a decrease in WLST rates and in‐hospital mortality independent of ICH score value.

## 2 Methods

### 2.1 Study Population

Our study utilized data from the Florida Stroke Registry (FSR), a statewide, ongoing quality improvement initiative which includes patients admitted with diagnoses of acute stroke across 181 participating hospitals in Florida. The registry prospectively collects standardized clinical, imaging, and outcome variables using the American Heart Association's Get With The Guidelines‐Stroke (GWTS‐S). Details of the FSR and data elements have been previously described [14].

This study was approved by the University of Miami's institutional review board (IRB). Participating centers were given institutional ethics approval to enroll patients in the registry; centers were not required to obtain individual patient consent under the common rule or waiver of authorization and exception from subsequent review by their IRB. The study was performed in accordance with the ethical standards on human experimentation as established in the Helsinki Declaration of 1975.

Using the registry data, we identified eligible patients with a primary diagnosis of ICH from 2013 to 2022. Patients were identified based on recorded final clinical diagnosis of “Intracerebral hemorrhage” from the GWTG‐S questionnaire. From this pool, patients with documented ICH scores were further identified, and patients without listed ICH scores were excluded. As key elements required to calculate the ICH score (such as hematoma volume, infratentorial location, and presence of intraventricular hemorrhage) were not systematically recorded for all patients, retrospective calculation of ICH scores for excluded cases was not feasible. Baseline characteristics were compared between patients with and without documented scores to assess for selection bias and ensure comparability between groups.

ICH scores were determined at initial presentation as a baseline severity score based on standard components (GCS, hematoma volume, presence of IVH, infratentorial origin, and age > 80 years), as previously described [13]. As ICH score ranges from 0 to 6, scores were stratified into three groups: ICH score of 0 to 2; ICH score of 3 to 4; and ICH score of 5 to 6. For patients identified to have a diagnosis of ICH, the decision for WLST during hospitalization was determined as a discrete data element based on having GWTG‐S documented responses indicating WLST or transition to comfort measures only. Early WLST was defined as a WLST decision occurring within 48 h of hospital presentation, whereas late WLST was defined as occurring more than 48 h after presentation, based on responses recorded in the GWTG‐S questionnaire [2, 6].

Data collected included demographic information (age, sex, race/ethnicity); insurance status (private, Medicare, Medicaid, self/no‐insurance, and unknown); information regarding stroke center type (comprehensive stroke center, thrombectomy capable stroke center, or primary stroke center); comorbidities (hypertension, diabetes, obesity, prior history of smoking, alcohol/drug use, atrial fibrillation or flutter, coronary artery disease, peripheral vascular disease, prior stroke, heart failure); prior ambulation status; receipt of intravenous thrombolysis or endovascular treatment; and surgical interventions (craniotomy or evacuation, external ventricular drain, multiple procedures or other). Measures of disease severity including initial ICH score and GCS score were also collected. Geographic region within Florida was categorized as east center, west central, north, panhandle, and south Florida. In‐hospital outcomes included mortality, ambulation status on discharge (independent or needs assistance), and discharge disposition (home/rehab or other).

### 2.2 Statistical Analysis

We first summarized baseline patient characteristics and clinical outcomes, followed by modeling the association between ICH score and WLST as well as mortality outcomes. Continuous variables were summarized as medians with first and third quartiles (Q1 and Q3). Descriptive statistics were performed using Pearson chi‐squared and Kruskal–Wallis tests.

Importance plots were generated using random forest (RF) models to identify predictors associated with WLST. Variable importance was determined based on the mean decrease in Gini impurity, a measure of how much each variable improved classification accuracy. The following factors were included in the RF model and importance plot: ICH score value, age, sex, race/ethnicity, insurance status, smoking, drug/alcohol use, hypertension, diabetes, obesity, atrial fibrillation/atrial flutter, coronary artery disease, peripheral vascular disease, prior stroke or TIA, prior ambulation, stroke center type, surgical treatment, state region, and impaired level of consciousness on presentation. Variables were selected based on clinical relevance and low missingness (< 5%).

Based on the most predictive variables identified in the performance plot, logistic regression (LR) and RF were performed to further evaluate predictors of WLST. Model performance for both the RF and LR models was assessed using receiver operating characteristic (ROC) curve analysis, with calculation of the area under the curve (AUC) for each model. The dataset was randomly divided into three subsets: 70% for training the model, 15% for validating model parameters, and 15% for final testing of model performance. This internal validation model approach was used to evaluate model accuracy and generalizability. Both models incorporated the top 10 predictors of WLST, including ICH score, age, geographic region, impaired level of consciousness on presentation, insurance status, race, surgical treatment type, sex, stroke center type, and history of diabetes.

Analyses of secondary outcomes were performed using univariate logistic regression to evaluate the following associations: ICH score and in‐hospital mortality; ICH score and overall WLST; and ICH score and early WLST. Analyses were restricted to data from 2015 to 2022, as early registry years (2013–2014) had low case numbers and limited documentation of ICH scores, precluding reliable analysis. ICH score groups were stratified as 0–2 (reference), 3–4, and 5–6. Stratified univariate logistic regression analyses were performed for two time periods (2015–2018 and 2019–2022) to assess temporal trends in associations. Models were adjusted for age, sex, race/ethnicity, comorbidities (hypertension, diabetes, obesity, prior stroke, or TIA), region, insurance status, and hospital type.

Associations were estimated with odds ratios (ORs) and 95% confidence intervals (CIs). Statistical significance was set at *p* < 0.05. Analyses were performed using SAS Version 9.4 software (SAS Institute Inc., Cary, NC), and R Version 4.2.2 (R Foundation for Statistical Computing, Vienna, Austria).

## 3 Results

### 3.1 Study Population

A total of 60,729 patients with a diagnosis of ICH were included in the FSR between 2013 and 2022. Of these patients, we studied a total of 12,426 (20%) who had documented ICH scores. Intergroup differences between ICH patients with and without documented ICH scores are shown in Table S1. Patients with documented ICH scores had similar rates of in‐hospital mortality compared to patients without documented scores (17% vs. 18%). WLST occurred in 27.3% of patients with a documented ICH score, compared to 24.7% of patients without a documented ICH score (*p* < 0.001). A greater proportion of patients without ICH scores had unknown WLST status (10.4%) compared to those with documented scores (< 0.01%) (Table S1).

Of the 12,426 patients with documented ICH scores included, median age was 69 (SD = 14.96); 45% were women, 56% were White, 24% were Black, and 20% Hispanic. Of these patients, 76% had hypertension, 25% had diabetes, 24% had obesity, and 23% had prior history of stroke or transient ischemic attack (TIA). Patients were seen primarily in comprehensive stroke centers (84%) and thrombectomy‐capable stroke centers (5%); median time from onset of symptoms to arrival time was 177 min (61, 515). More detailed characteristic data stratified by ICH score group are shown in Table 1.

**TABLE 1:** Baseline characteristics of ICH patients from 2013 to 2022 stratified by ICH score group.

Variables	Overall, *n* = 12,425	ICH score 0–2, *n* = 8630	ICH score 3–4, *n* = 3214	ICH score 5–6, *n* = 581
Age, median (IQR)	69 (57, 79)	68 (57, 77)	72 (58, 82)	80 (63, 85)
Sex, *n* (%)				
Male	6780 (54.57%)	4835 (56.03%)	1677 (52.18%)	268 (46.13%)
Female	5645 (45.43%)	3795 (43.97%)	1537 (47.82%)	313 (53.87%)
Race/ethnicity, *n* (%)				
White	6997 (56.31%)	4776 (55.34%)	1878 (58.43%)	343 (59.04%)
Black	2979 (23.98%)	2136 (24.75%)	734 (22.84%)	109 (18.76%)
Hispanic	2449 (19.71%)	1718 (19.91%)	602 (18.73%)	129 (22.20%)
Insurance status, *n* (%)				
Private	3209 (25.83%)	2291 (26.55%)	811 (25.23%)	107 (18.42%)
Medicare	6033 (48.56%)	4033 (46.73%)	1647 (51.24%)	353 (60.76%)
Medicaid	794 (6.39%)	553 (6.41%)	210 (6.53%)	31 (5.34%)
Self/no insurance	2389 (19.23%)	1753 (20.31%)	546 (16.99%)	90 (15.49%)
Smoking, *n* (%)	1391 (11.20%)	1081 (12.53%)	275 (8.56%)	35 (6.02%)
Drug/alcohol use, *n* (%)	1216 (9.79%)	889 (10.30%)	291 (9.05%)	36 (6.20%)
Hypertension, *n* (%)	9469 (76.21%)	6609 (76.58%)	2398 (74.61%)	462 (79.52%)
Diabetes, *n* (%)	3076 (24.76%)	2145 (24.86%)	802 (24.95%)	129 (22.20%)
Obesity, *n* (%)	3005 (24.19%)	2179 (25.25%)	713 (22.18%)	113 (19.45%)
Atrial fibrillation/atrial flutter, *n* (%)	1961 (15.78%)	1271 (14.73%)	574 (17.86%)	116 (19.97%)
Coronary artery disease, *n* (%)	1851 (14.90%)	1229 (14.24%)	507 (15.77%)	115 (19.79%)
Peripheral vascular disease, *n* (%)	364 (2.93%)	253 (2.93%)	96 (2.99%)	15 (2.58%)
Prior stroke or TIA, *n* (%)	2862 (23.03%)	1965 (22.77%)	771 (23.99%)	126 (21.69%)
Stroke center type, *n* (%)				
CSC	10,458 (84.17%)	7251 (84.02%)	2711 (84.35%)	496 (85.37%)
PSC	1384 (11.14%)	988 (11.45%)	344 (10.70%)	52 (8.95%)
TSC	583 (4.69%)	391 (4.53%)	159 (4.95%)	33 (5.68%)
Onset of symptoms to arrival time in minutes, median (IQR)	177 (61, 515)	182.00 (62.00, 541.00)	162.00 (60.00, 454.00)	149.00 (60.00, 453.00)
Door to CT Time in minutes, median (IQR)	19 (10, 52)	18 (10, 62)	21 (11, 43)	26 (15, 46)
Region, *n* (%)				
South	5372 (43.24%)	3732 (43.24%)	1351 (42.03%)	289 (49.74%)
West Central	2454 (19.75%)	1652 (19.14%)	680 (21.16%)	122 (21.00%)
East Central	1568 (12.62%)	1125 (13.04%)	385 (11.98%)	58 (9.98%)
North	2340 (18.83%)	1613 (18.69%)	628 (19.54%)	99 (17.04%)
Panhandle	691 (5.56%)	508 (5.89%)	170 (5.29%)	13 (2.24%)
Impaired level of consciousness, *n* (%)	3915 (31.51%)	1917 (22.21%)	1661 (51.68%)	337 (58.00%)
In‐hospital mortality, *n* (%)	2129 (17.13%)	487 (5.64%)	1257 (39.11%)	385 (66.27%)
Hospital length of stay in days, median (IQR)	6.24 (3.17, 12.06)	6.84 (3.92, 12.04)	5.45 (2.03, 13.84)	1.89 (0.95, 4.13)
WLST, *n* (%)	3393 (27.31%)	1097 (12.71%)	1873 (58.28%)	423 (72.81%)
Early WLST (within 48 h), *n* (%)	1283 (10.33%)	211 (2.44%)	777 (24.18%)	295 (50.77%)
Discharge disposition, *n* (%)				
Home/rehab	5759 (46.35%)	5324 (61.69%)	414 (12.88%)	21 (3.61%)
Other	6666 (53.65%)	3306 (38.31%)	2800 (87.12%)	560 (96.39%)

*Note:* Data are presented as *n* (%) or median (Q1–Q3).

Abbreviations: CSC, Comprehensive Stroke Center; GCS, Glasgow Come Scale; ICH, Intracerebral hemorrhage; PSC, Primary Stroke Center; TIA, Transient Ischemic Attack; TSC, Thrombectomy‐Capable Stroke Center; WLST, withdrawal of life‐sustaining treatment.

A total of 8631 (69%) of patients had an ICH score of 0–2, 3214 (26%) patients had an ICH score of 3–4, and 581 (5%) of patients had an ICH score of 5–6. Overall, in‐hospital mortality was observed in 2129 (17%) of all patients, with an average hospital length of stay of 6.25 days (SD = 14). The in‐hospital mortality rate for ICH scores 0–2 was 6.6%, 41.5% for scores 3–4, and 66% for scores 5–6.

The decision to WLST was seen in 3393 (27%) patients overall. WLST occurred in 12.7% of patients with ICH scores 0–2, 58.3% of patients with scores 3–4, and 72.8% of patients with scores 5–6. Early WLST occurred in 2.4% of patients with scores 0–2, 24.2% of patients with scores 3–4, and 50.8% of patients with scores 5–6. Among patients who died during hospitalization (*n* = 2129), 1556 (73.1%) patients had WLST documented (Table 1).

### 3.2 Model Performance for Predicting WLST Decision

**FIGURE 1:**
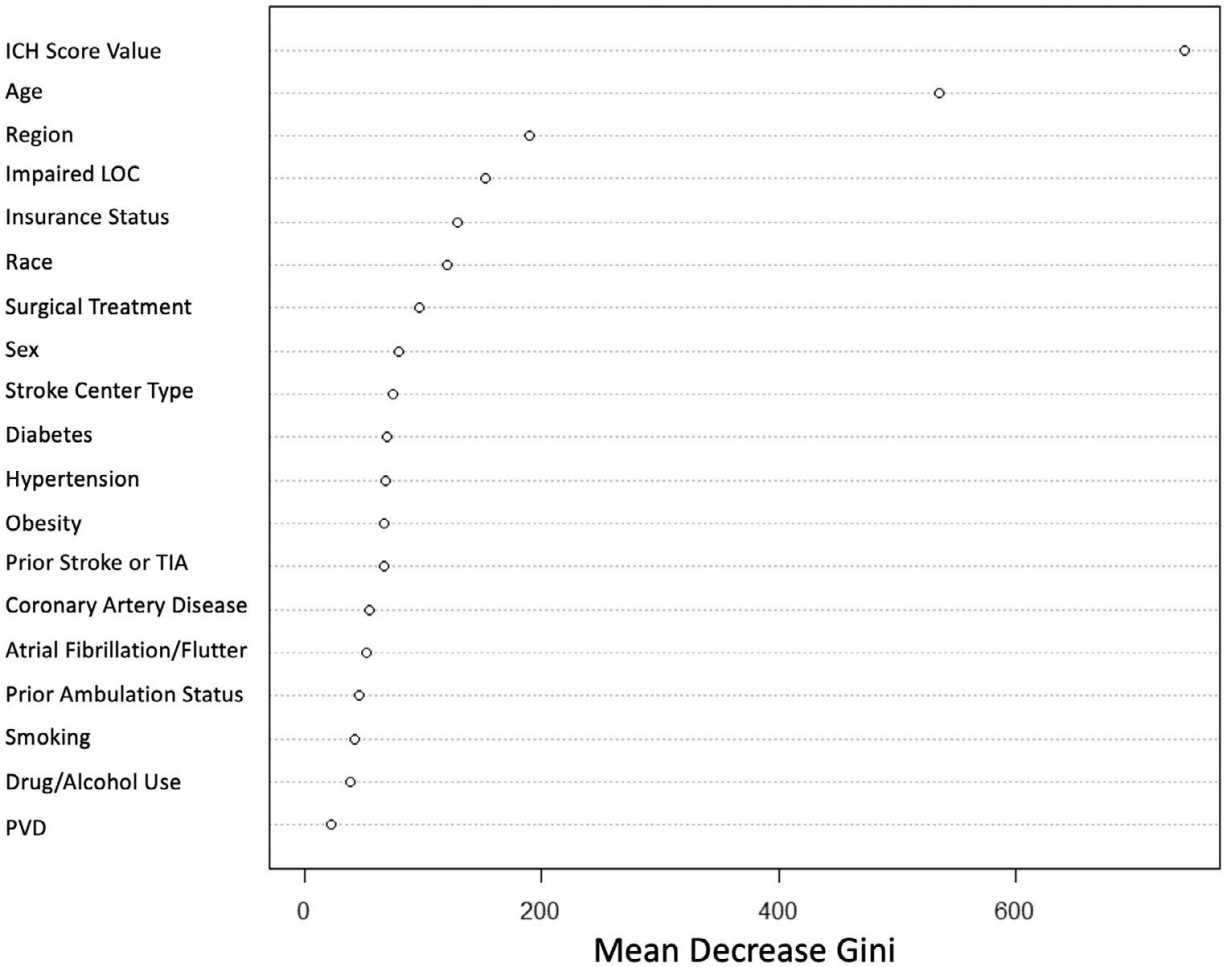
Variable importance plot for predictors of WLST decision in random forest model. ICH, intracerebral hemorrhage; Impaired LOC, impaired loss of consciousness on presentation; PVD, peripheral vascular disease; TIA, transient ischemic attack; WLST, withdrawal of life‐sustaining treatment. Variable importance ranked by mean decrease in Gini index.

**FIGURE 2:**
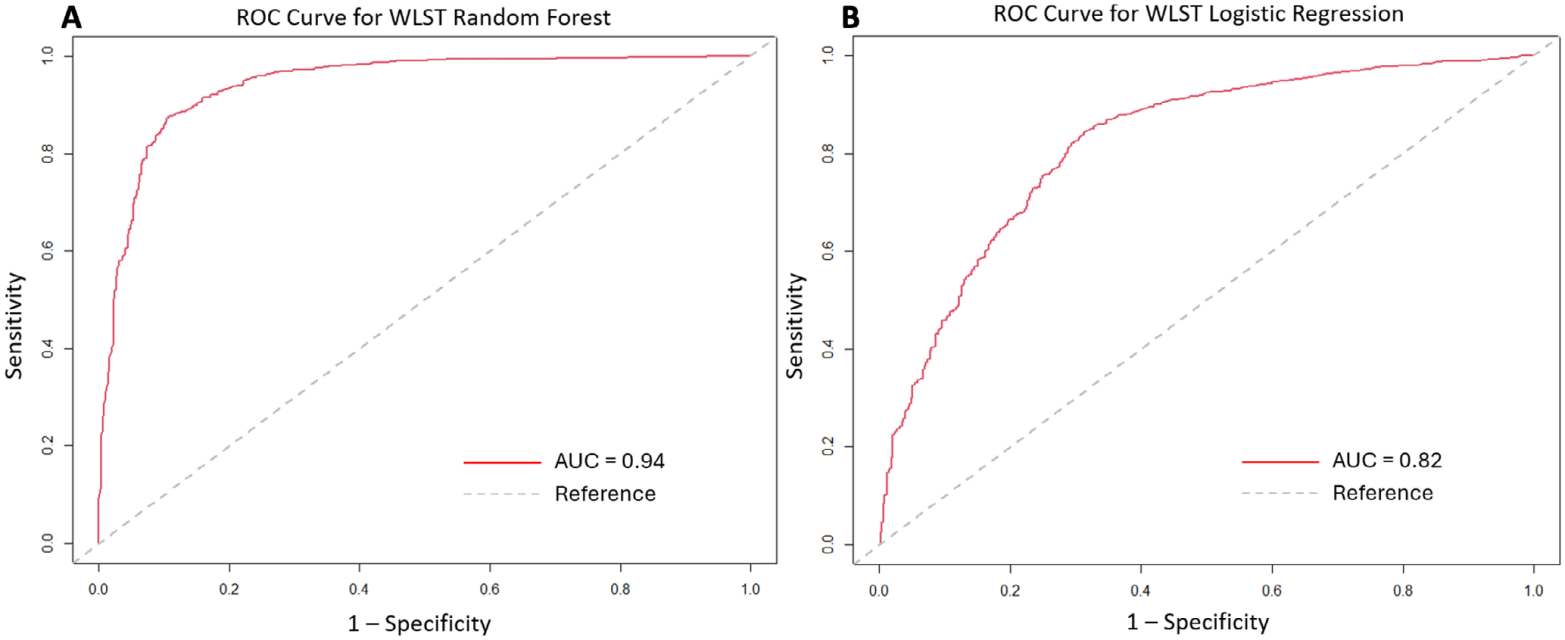
Receiver operator characteristic (ROC) curves for predictors of WLST decision. (A) ROC curve for random forest model; (B) ROC curve for logistic regression model. AUC indicates area under the curve; ROC, receiver operating characteristic; WLST, withdrawal of life‐sustaining treatment.

A total of 12,425 individuals with documented ICH scores and WLST outcomes were included in the analysis. Variable importance was ranked using the Mean Decrease in Gini metric, with ICH score value, age, region, impaired level of consciousness on presentation, insurance status, race, surgical treatment type, sex, stroke center type, and diabetes emerging as the top predictors (Figure 1). The most predictive factor associated with WLST decision was ICH score value. The RF model demonstrated excellent discrimination with an AUC of 0.94, and the LR model showed strong discrimination with an AUC of 0.82 (Figure 2).

### 3.3 Mortality and WLST Decision by ICH Score Group

Secondary outcomes were analyzed using data from 2015 to 2022 due to limited patient case numbers in the earlier registry years (2013–2014). Increasing ICH scores were significantly associated with higher odds of in‐hospital mortality, overall WLST, and early WLST decisions. Compared to patients with ICH scores of 0–2, patients with higher scores had markedly increased odds of mortality and WLST. Higher ICH scores were also associated with a greater likelihood of early WLST, defined as occurring within 48 h of hospital presentation. A statistically significant stepwise increase in both overall WLST and early WLST was observed across score groups. Full ORs, 95% CIs, and *p* values for these associations are presented in Table 2.

**TABLE 2:** Univariate logistic regression for mortality and WLST by ICH score group and time period, 2015 to 2022.

Outcome	ICH score group	Overall		2015–2018		2019–2022	
		OR	95% CI	OR	95% CI	OR	95% CI
Mortality	0–2	Reference	—	Reference	—	Reference	—
	3–4	10.69	9.49–12.04	11.90	9.67–14.65	10.11	8.75–11.68
	5–6	34.40	28.14–42.04	46.85	32.05–68.47	30.24	23.82–38.38
WLST	0–2	Reference	—	Reference	—	Reference	—
	3–4	9.35	8.50–10.30	9.90	8.28–11.84	9.30	8.29–10.43
	5–6	18.64	15.28–22.74	16.25	11.61–22.76	20.67	16.10–26.53
Early WLST	0–2	Reference	—	Reference	—	Reference	—
	3–4	2.97	2.48–3.55	2.98	2.11–4.22	2.95	2.39–3.63
	5–6	9.51	7.33–12.35	10.08	6.07–16.75	9.28	6.84–12.58

*Note:* Early WLST refers to WLST decision made within 48 h of hospital presentation. The reference group for all outcomes is ICH score 0–2, which is assigned as an odds ratio of 1.0. Models were adjusted for age, sex, race/ethnicity, comorbidities (hypertension, diabetes, obesity, prior stroke, or TIA), region, insurance status and hospital type. All ORs for each ICH score group and time period were statistically significant with *p* < 0.0001.

Abbreviations: ICH, Intracerebral Hemorrhage; WLST, Withdrawal of Life‐Sustaining Treatment.

### 3.4 Temporal Differences in Mortality by ICH Score Group

**FIGURE 3:**
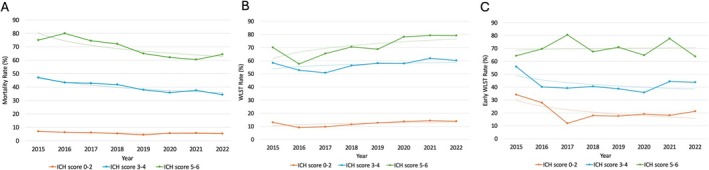
Temporal trends in mortality and WLST by ICH score group. (A) Mortality rates by ICH score group from 2015 to 2022. (B) WLST rates by ICH score group from 2015 to 2022. (C) Rates of early WLST by ICH score group from 2015 to 2022, expressed as a percentage of total WLST decisions within each group. ICH indicates intracerebral hemorrhage; WLST, withdrawal of life‐sustaining treatment. Early WLST refers to the WLST decision made within 48 h of hospital presentation.

Stratified univariate logistic regression analyses were performed for the 2015–2018 and 2019–2022 periods. Higher ICH scores remained significantly associated with increased mortality across both time periods. Although the odds of mortality were slightly lower in the 2019–2022 cohort than the 2015–2018, patients with higher ICH scores continued to have substantially elevated mortality risk relative to those with scores of 0–2. Full ORs, CIs, and *p* values are presented in Table 2. Temporal trends in mortality by ICH score group are illustrated in Figure 3A.

### 3.5 Temporal Differences in WLST by ICH Score Group

Higher ICH scores were strongly associated with increased odds of WLST across both examined time periods. In both the 2015–2018 and 2019–2022 cohorts, patients with ICH scores of 3–4 and 5–6 consistently demonstrated a significantly greater likelihood of WLST compared to those with lower scores. A similar pattern was observed for early WLST, which remained significantly more likely in patients with higher ICH scores. Full ORs, CIs, and *p* values are presented in Table 2. Temporal trends in overall WLST and early WLST by ICH score group can be seen in Figure 3B,C.

## 4 Discussion

In this large, prospective statewide stroke registry of patients with ICH between 2013 and 2022, we found that higher ICH scores were strongly associated with in‐hospital mortality, WLST, and early WLST decisions. WLST occurred more frequently in patients with higher ICH score values, and early WLST was more common among those with higher ICH scores. Although rates of mortality did appear somewhat lower in more recent years, the strong association between ICH score and both WLST and mortality persisted across the study period. Among patients who died during hospitalization, 73.1% had WLST documented, highlighting the strong relationship between WLST and mortality in this cohort. Other factors associated with WLST included age, insurance status, race, geographic region, and impaired level of consciousness. These findings align with prior studies showing that socioeconomic status and other social determinants of health have been noted to be associated with WLST decisions after ICH [15]. Nevertheless, ICH score remained the most predictive variable overall.

These findings raise important considerations regarding how early prognostic assessments may shape treatment decisions. Unlike the modeling used by Becker et al. [4] to compare predicted versus observed outcomes under early care limitations, our study is observational and based on data from a large, multicenter stroke registry. The ICH score grading scale was initially proposed to provide information and assess treatment benefits and risks for patient families, rather than being used to directly prognosticate outcomes [13, 16]. Since its initial publication, there has been a tendency by healthcare providers to use this scale as a prognostication tool to influence clinical decision‐making [8, 17, 18]. Like other severity scales in neurocritical care, this may contribute to a self‐fulfilling prophecy where perceived futility by the medical team leads to early care decisions [6, 11, 19, 20].

ICH is associated with high morbidity and mortality, and in addition often with higher rates of WLST than other disease processes within neurocritical care, making it difficult to disentangle mortality from the influence of care decision‐making [11, 20, 21, 22]. Recent FSR data from 2008 to 2021 demonstrated that WLST decisions occurred in 9% of acute ischemic stroke patients, 28% in ICH, and 19% in SAH [2]. Given the nihilism surrounding prognosis historically, it is not surprising that ICH score was identified as the most predictive variable associated with WLST.

Relevant guidelines published during the study period also warrant consideration. American Heart Association/American Stroke Association (AHA/ASA) guidelines published in 2010 highlight the uncertainty and potential for self‐fulfilling prophecies of poor outcome with use of ICH score [23]. At the time, guidelines suggested avoidance of recommendations of care limitations such as do not resuscitate (DNR) orders or WLST within at least the second full day of hospitalization [23, 24, 25]. A study from 2015 observed that patients without placement of DNR orders within the first 5 days after ICH presentation had a substantially lower 30‐day mortality than predicted by the ICH score [26]. That same year, AHA/ASA guideline updates emphasized postponing DNR or WLST in the absence of pre‐existing advanced directives until at least the second full day of hospitalization [27]. Additionally, 2015 guidelines advised that DNR orders should not limit appropriate medical and surgical interventions [28]. Most recent guidelines continue to make these practice recommendations [29, 30].

Despite these guideline recommendations made during the corresponding time frames, we observed a continued association between higher ICH scores and WLST decision. While only a proportion of WLST does appear to occur early, we found a persistent association between ICH score value and early WLST, without a notable change between the two time periods in the study. Although early WLST was less frequent than WLST overall, patients with higher ICH scores were significantly more likely to experience early withdrawal than those with lower scores.

Improvement in mortality over time is not surprising given efforts over time to improve and bundle care with respect to prompt blood pressure lowering, anticoagulant reversal, and improved neurosurgical access, among other efforts [31]. Additionally, minimally invasive hematoma evacuation has been recently demonstrated to result in improvement in mortality and functional outcome [32, 33].

The COVID‐19 pandemic may have influenced mortality over the 2019 to 2022 time period [34, 35, 36]. Moreover, resource limitations and substantial comorbidities during that time could have impacted care decisions [37]. Data regarding COVID‐19 testing or comorbid infection were collected in our registry. Nonetheless, assuming a continued trajectory toward advances in therapeutics for ICH, we anticipate both a continued trend toward decreased mortality and a potential shift in favor of a more aggressive approach to treatment over time.

Decisions regarding WLST have limited understanding of recovery in this disease process [22]. There is a paucity of high‐quality data investigating outcomes in ICH beyond 3–6 months following initial presentation [9, 38, 39]. In a large cohort of over 500 ICH patients from a prospective registry over 5 years of patients who were maximally treated, there was a long‐term mortality rate of 30%, and 45% of patients reached a favorable functional outcome by modified Rankin Scale (mRS 0–3) at 12‐month follow up [8]. A subsequent multicenter validation study compared outcome prognostication of the max‐ICH score, which excludes patients without early care limitations, to that of the ICH score, and demonstrated improved prognostication of functional outcome for such patients [17]. Less still has been published on longer term outcome trajectories in ICH patients, although one such study demonstrated that among those with mRS of 4–5 at 30‐day following ICH, over 40% recovered to a favorable functional outcome (mRS 0–3) at 1 year [40].

There are limitations with respect to our study. Firstly, important clinical information—including anticoagulant or antiplatelet use, code status, prior advanced directives, and extent to which medical therapies were continued or withheld—was not consistently available in the FSR and therefore could not be considered. In addition, details regarding WLST decision‐making and the extent to which this was influenced by patient and family‐centered views were also not available. ICH score documentation was incomplete, though the 12,426 patients with scores had broadly similar characteristics to those without. Notably, WLST rates were slightly higher among patients with documented ICH scores compared to those without, and a substantial proportion of WLST status was missing among patients without ICH scores, raising the possibility of a selection bias. It is also possible that the formal documentation of an ICH score may prompt a greater clinical focus on prognostication and end‐of‐life decision‐making. Given the observational nature of our study, only associations could be evaluated, and causality cannot be inferred. Additionally, the FSR does not include data on cognitive and functional outcomes after hospitalization, making it difficult to fully assess the impact of the self‐fulfilling prophecy in this population over time.

Future directions should aim to better infer causal relationships between initial ICH severity scores, WLST decisions, and outcomes. Prospective cohort studies that standardize the timing of early prognostic and goals‐of‐care discussions—for example, developing formal protocols to delay these conversations until after 48–72 h—could help reduce bias from early prognostic pessimism. A stepped‐wedge cluster randomized design could evaluate the impact of implementing structured communication pathways or delayed‐decision protocols across different sites over time, helping to disentangle the effects of early prognostication on patient outcomes [41].

WLST in ICH remains common, particularly for perceived high severity patients. Despite efforts to shift away from early WLST, particularly based on ICH score severity, ICH score continues to be the most predictive variable associated with WLST, and the decision to WLST, particularly within the first 48 h, also continues to occur. These findings raise concerns about a potential persistence of the self‐fulfilling prophecy framework in ICH. Because WLST is a discrete clinical decision—rather than a continuously graded probability—it is particularly vulnerable to influence by provider perceptions, patient‐family dynamics, and system‐level factors. Recognizing and addressing these ongoing concerns is crucial to preventing premature treatment decisions and ensuring more informed and unbiased care for ICH patients.

## Author Contributions

N.M., A.A., N.A., and H.G. were involved in the study concept and design. L.Z., H.Y., C.M.G., and H.G. were involved in the acquisition of data. L.Z., B.M., and H.G. were involved in the statistical analysis. L.Z., B.M., N.M., A.A., and H.G. were involved in the analysis and interpretation of data. N.M., A.A., L.Z., B.M., N.A., H.G., H.Y., C.M.G., A.J., D.R., M.K., A.M., K.O., S.K., J.G.R., and T.R. were involved in drafting the article. N.M., A.A., C.M.G., and N.A. were involved in study supervision.

## Disclosure

Sources of funding: The Florida Stroke Registry (FSR) is a comprehensive repository of data collected from 181 participating hospitals statewide; its aim is to identify disparities in stroke care and develop interventions to improve the quality of stroke care in Florida. The registry was initially funded by the National Institute of Health/National Institute of Neurological Disorders, then known as the Florida‐Puerto Rico Collaboration to Reduce Stroke Disparities, and since 2017 has continued through funding support from the state of Florida (COHAN‐A1).

## Conflicts of Interest

A.A. is supported by an institutional KL2 Career Development Award from the Miami CTSI NCATS UL1TR002736, institutional ULink award, and by the National Institute of Neurological Disorders and Stroke of the National Institutes of Health under Award Number K23NS126577, R21NS128326, and R21NS136970. He is also supported by DoD grant #BA230159. N.A. is supported by salary support from the Florida Stroke Registry (FSR) COHAN‐A1 R2 contract. T.R. is funded by the Florida Department of Health for work on the FSR and by the grants from the NIH (R01 MD012467, R01 NS29993, R01 NS040807, and 1U24 NS107267), and the NCATS (UL1 TR002736 and KL2 TR002737). The other authors declare no conflicts of interest.

## Supporting information


**Table S1.** Selected baseline characteristics and outcomes of patients with and without documented ICH scores (2013–2022).

## Data Availability

The FSR uses data from the American Heart Association (AHA) Get With the Guideline‐Stroke (GWTG‐S). As GWTG‐S is collected primarily for quality improvement, data‐sharing agreements require an application process for other researchers to access data. Research proposals can be submitted at www.heart.org/qualityresearch and will be considered by the GWTG‐S and the FSR advisory and publication committees upon reasonable request.
