# Supplementary material for: Association of the ICH Score With Withdrawal of Life‐Sustaining Treatment Over a 10‐Year Period
Source: Ann Clin Transl Neurol. 2025 Jul 2;12(10):1992–2001. doi: 10.1002/acn3.70136 (PMC12516225; doi:10.1002/acn3.70136)
Supplement: Supplementary file 1 — Table S1. Selected baseline characteristics and outcomes of patients with and without documented ICH scores (2013–2022). [file ACN3-12-1992-s001.docx]

Supplementary Table 1. Selected Baseline Characteristics and Outcomes of Patients With and Without Documented ICH Scores (2013-2022)

|  |  | **Overall**  **ICH Patients N=60729** | **With ICH score N=12426** | **Without ICH score N=48303** | **P-Value** |
| --- | --- | --- | --- | --- | --- |
| Age (SD) |  | 71 (15.19) | 69 (14.96) | 72 (15.23) | <.001 |
| Sex, n (%) | Male | 32223 (53.09%) | 6780 (54.56%) | 25443 (52.71%) | <.001 |
|  | Female | 28476 (46.91%) | 5646 (45.44%) | 22830 (47.29%) |  |
| Race, n (%) | White | 40185 (66.17%) | 6997 (56.31%) | 33188 (68.71%) | <.001 |
|  | Black | 12201 (20.09%) | 2980 (23.98%) | 9221 (19.09%) |  |
|  | Hispanic | 8343 (13.74%) | 2449 (19.71%) | 5894 (12.20%) |  |
| Insurance Status, n (%) | Private | 16152 (26.60%) | 3209 (25.82%) | 12943 (26.80%) | <.001 |
|  | Medicare | 25086 (41.31%) | 6034 (48.56%) | 19052 (39.44%) |  |
|  | Medicaid | 3052 (5.03%) | 794 (6.39%) | 2258 (4.67%) |  |
|  | Self/No Insurance | 8566 (14.11%) | 2389 (19.23%) | 6177 (12.79%) |  |
|  | Missing | 7873 (12.96%) | 0 (0.00%) | 7873 (16.30%) |  |
| Smoking, n (%) |  | 7115 (11.72%) | 1391 (11.19%) | 5724 (11.85%) | 0.043 |
| Drugs or Alcohol Use, n (%) |  | 3396 (5.59%) | 1216 (9.79%) | 2180 (4.51%) | <.001 |
| Hypertension, n (%) |  | 43126 (71.01%) | 9469 (76.20%) | 33657 (69.68%) | <.001 |
| Diabetes, n (%) |  | 14461 (23.81%) | 3076 (24.75%) | 11385 (23.57%) | 0.006 |
| Obesity, n (%) |  | 7354 (12.11%) | 3005 (24.18%) | 4349 (9.00%) | <.001 |
| Atrial fibrillation/Atrial flutter, n (%) |  | 10031 (16.52%) | 1961 (15.78%) | 8070 (16.71%) | 0.013 |
| Coronary Artery Disease, n (%) |  | 10836 (17.84%) | 1851 (14.90%) | 8985 (18.60%) | <.001 |
| Peripheral Vascular Disease, n (%) |  | 1686 (2.78%) | 364 (2.93%) | 1322 (2.74%) | 0.244 |
| Prior Stroke or TIA, n (%) |  | 12189 (20.07%) | 2862 (23.03%) | 9327 (19.31%) | <.001 |
| Stroke Center Type, n (%) | CSC | 34024 (56.03%) | 10459 (84.17%) | 23565 (48.79%) | <.001 |
|  | PSC | 23637 (38.92%) | 1384 (11.14%) | 22253 (46.07%) |  |
|  | TSC | 2856 (4.70%) | 583 (4.69%) | 2273 (4.71%) |  |
|  | Other | 212 (0.35%) | 0 (0.00%) | 212 (0.44%) |  |
| Region, n (%) | South | 20439 (33.66%) | 5372 (43.23%) | 15067 (31.19%) | <.001 |
|  | West Central | 18390 (30.28%) | 2454 (19.75%) | 15936 (32.99%) |  |
|  | East Central | 10841 (17.85%) | 1568 (12.62%) | 9273 (19.20%) |  |
|  | North | 6471 (10.66%) | 2341 (18.84%) | 4130 (8.55%) |  |
|  | Panhandle | 4582 (7.54%) | 691 (5.56%) | 3891 (8.06%) |  |
| Impaired Level of Consciousness, n (%) |  | 16735 (27.56%) | 3915 (31.51%) | 12820 (26.54%) | <.001 |
| Hospital Length of Stay in days (SD) |  | 5.13 (13.12) | 6.25 (14.01) | 4.88 (12.80) | <.001 |
| In-hospital Mortality, n (%) | Yes | 10900 (17.95%) | 2129 (17.13%) | 8771 (18.16%) | <.001 |
|  | No | 49206 (81.03%) | 10224 (82.28%) | 38982 (80.70%) |  |
|  | Missing | 623 (1.03%) | 73 (0.59%) | 550 (1.14%) |  |
|  | | | | | |
| WLST, n (%) | Yes | 15331 (25.24%) | 3393 (27.31%) | 11938 (24.71%) | <.001 |
|  | No | 40361 (66.46%) | 9032 (72.69%) | 31329 (64.86%) |  |
|  | Missing | 5037 (8.29%) | 1 (0.01%) | 5036 (10.43%) |  |
|  | | | | | |

Selected baseline demographic, clinical, and outcome characteristics comparing patients with documented ICH scores (n = 12,426) and those without documented ICH scores (n = 48,303). Variables with >20% missing data were excluded. Insurance status was missing in 16.3% of patients without documented ICH scores and was complete in patients with documented ICH scores; percentages for insurance categories are based on available data.

ICH indicates Intracerebral hemorrhage; WLST, withdrawal of life-sustaining treatment; TIA, Transient Ischemic Attack; CSC, Comprehensive Stroke Center; PSC, Primary Stroke Center; TSC, Thrombectomy-Capable Stroke Center.

*Data are presented as n (%) or median (Q1-Q3).
